# Assessment of chloride levels on renal function after cardiac arrest

**DOI:** 10.1186/2197-425X-3-S1-A194

**Published:** 2015-10-01

**Authors:** C Righy Shinotsuka, P Caironi, P Villois, V Fontana, JL Vincent, J Creteur, FS Taccone

**Affiliations:** Erasme University Hospital, Bruxelles, Belgium; University of Milano. Fondazione IRCCS Ca' Granda Ospedale Maggiore Policlinico, Milano, Italy

## Introduction

Chloride may alter renal function by inducing vasoconstriction of renal afferent arterioles, thereby inducing cortical hypoperfusion. Increased chloride delivery to the distal tube may also induce tubular dysfunction. The effects of chloride balance on renal function after cardiac arrest (CA) remain unknown.

## Objectives

To investigate whether chloride serum and urinary levels as well as intravenous chloride load, balance and the anion urinary gap (AG_U_) in the first day after CA are associated with the development of AKI.

## Methods

Retrospective analysis of an institutional database including all adult comatose patients admitted to the Intensive Care Unit (ICU) after CA (2007-2014). Inclusion criteria were age ≥18; non-traumatic arrest; complete data available on serum and urinary samples for at least 3 days; survival ≥ 72 hours after admission. Patients with previous AKI, with anuria on ICU admission or on chronic hemodialysis were excluded. Demographics, CA-related findings and outcome information were included in the database. We collected chloride levels on blood (BCl) and urinary (UCl) analyses on ICU admission; chloride load (CL) was calculated by considering the amount of chloride present in the IV fluids administered within the first 24 hours to the patient, while chloride balance (CB) was calculated as: CL - CO, where CO is chloride output (UCl * daily urine output). Creatinine clearance (CrCl) was calculated on 24-hr urinary collection. AKI was defined according to standard AKIN criteria.

## Results

Of the 404 eligible patients, 102 met the inclusion criteria. Median age was 58 [49-74] years and 75 (70%) were male. ICU mortality was 29% (n = 30). Forty-three patients (42%) developed AKI, after a median of 2 [2-4] days since admission (13 needed continuous renal replacement therapy). Patients with AKI had similar serum creatinine on admission and 24-hr fluid balance than others; however, 24-hr urine output (1111 [448-1870] vs. 2130 [1125-2775] mL, p = 0.004) and CrCl (21 [11-44] vs. 104 [71-143] mL/min; p < 0.001) were lower in patients with than without AKI. Although the initial BCl (102 [100-104] vs. 104 [101-107] mEq/L) and CL (341 [160-504] vs. 315 [201-436]) were similar between groups, patients with AKI had a lower UCl (66 [33-95] vs. 106 [61-138] mEq/L; p = 0.001) and CO (62 [11-125] vs. 177 [96-244] mEq; p < 0.001) than patients without AKI. This resulted in a higher CB (221 [68-462] vs. 125 [6-296] mEq; p = 0.02) and a trend towards higher (AG_U_) (45 [32-66] vs. 37 [24-54]; p = 0.16) in AKI patients when compared to others.

## Conclusions

After CA, AKI occurred in 40% of patients and was associated with a significant alteration in renal chloride elimination, already during the first day. The pathological role of chloride and the impact of such findings on the management of fluid therapy in this setting remain to be further evaluated.

